# Ultrasensitive Pressure Measurement Using an Extrinsic Fabry–Pérot Interferometer (EFPI) Sensor

**DOI:** 10.3390/s25185757

**Published:** 2025-09-16

**Authors:** Anthony Weir, Ben Bartlett, Gerard Dooly, Dinesh Babu Duraibabu

**Affiliations:** 1 Center for Robotics and Intelligent Systems (CRIS), Department of Electronic and Computer Engineering, University of Limerick, V94 T9PX Limerick, Ireland; ben.bartlett@ul.ie (B.B.); gerard.dooly@ul.ie (G.D.); 2 School of Engineering, University of Limerick, V94 T9PX Limerick, Ireland; 3 Department of Mechatronic Engineering, Atlantic Technological University, Ash Ln, Ballytivnan, F91 YW50 Sligo, Ireland

**Keywords:** optical fibre sensor, extrinsic Fabry–Pérot interferometry, diaphragm, pressure sensor

## Abstract

This paper advances the development of Extrinsic Fabry–Pérot interferometry (EFPI) for high-precision pressure sensing. Presented is an EFPI featuring a diameter of 800 μm with a 7.4 μm diaphragm thickness, demonstrating a resolution of 3.35 mPa and a sensitivity of 149 nm/kPa positioning it amongst the most sensitive fibre optic pressure sensors ever developed, establishing a new benchmark for EFPI pressure-based systems. Numerous fabrication methods, including resin bonding, fusion splicing, and additive manufacturing, are investigated. In conjunction with this, multiple diaphragm reduction techniques such as manual polishing, automated polishing, and hydrofluoric acid etching are explored. The reason why we have not seen development of large core/diameter silica EFPI sensors, with advantages in sensitivity and resolution, is that the construction technique is difficult and unknown. The design construction, testing, and development of said large-diameter sensor is novel. This sub-Pascal resolution opens new possibilities for applications in microfluidics, atmospheric monitoring, and medical diagnostics where detecting minute pressure variations is critical. Finally, a comparative analysis of the sensor construction and diaphragm reduction methods provides insight into the future development of these high-performance EFPI sensors.

## 1. Introduction

High-precision pressure sensing represents a critical measurement capability throughout numerous scientific and industrial domains. The ability to detect minuscule pressure variations at sub-Pascal level enables advanced applications in atmospheric monitoring, aerospace systems, microfluidic control, and medical diagnostics. Conventional electronic pressure sensors face significant limitations in environments with electromagnetic interference, high temperatures, or where miniaturization is essential. In the scenario where drones may be navigating without GPS, precise altitude control will rely on sub-Pascal resolution from barometric sensors [1].

Extrinsic Fabry–Pérot Interferometry (EFPI) offers distinct advantages for minute pressure measurement. These sensors utilize the principles of light interference within a precisely controlled cavity, where minuscule pressure-induced deflections of a diaphragm modulate the optical signal. Fabry and Pérot [2] introduced this in 1897. This interferometric technique has been universally applied to pressure-sensing applications. The non-electrical nature of signal transmission, immunity to electromagnetic interference, miniature dimensions, and capacity for remote sensing make EFPI sensors ideal for difficult measurement environments.

Sub-Pascal accuracy and practical manufacturability remain challenging. Most existing designs compromise between sensitivity and robustness or require complex fabrication techniques [3]. Various construction methods and materials have been explored, including electrical arc discharge techniques demonstrated by Liu et al. [4] with diaphragms as thin as 170 nm, and alternative materials such as epoxy adhesive films studied by Zhang et al. [5] and polymeric diaphragms by Eom et al. [6]. The compromise between diaphragm dimensions, material properties, and fabrication methods directly relates to sensor performance characteristics, including sensitivity, measurement range, and long-term stability [7].

Fabry and Pérot first introduced interferometry in 1897, and it has been used extensively within the role of pressure sensing [8,9]. Sensor immunity to electromagnetic interference coupled with its minute dimensions makes it a viable option for medical applications [10,11,12]. For example, Sato et al. study aimed to achieve grasp force measurement for forceps utilized in minimally invasive surgical robots [13]. The EFPI has also been utilized in a wide range of industry environments. Kienitz et al. developed an EFPI pressure sensor for use on an aircraft wing [14]. Liu et al. demonstrated a pressure based on an EFPI utilizing a fibre-tipped diaphragm-sealed cavity (FDC). The sensor used a generated diaphragm of 170 nm, using an optimized electrical arc discharge technique [4].

Li et al. constructed a silican EFPI with a diaphragm diameter of 3600 μm and a thickness of 200 μm with a sensitivity of 3410 nm/kPa [15].

The electrical arc discharge charge method of construction was demonstrated by Liao et al. [16] and Liu et al. [4] with respective sensitivities of 1.036 nm and 12.22 nm.

Wu et al. demonstrated an 11 μm thick 3D-printed diaphragm with a diameter of 80 μm and a ceramic ferrule, achieving a sensitivity of 3.3 nm/kPa.

Other materials have been investigated, such as Cheng et al. silk fibroin, Eom et al. epoxy polymeric diaphragm, and Zhang et al. epoxy adhesive film. Other silica diaphragms have been constructed with diaphragms similar to the diameter of an SMF fibre [17,18,19].

Other MEMS pressure sensing principles are utilized for sensing, such as piezocapacitive units. Balderrama et al. [20] simulated a piezoresistive sensor. The diaphragm consists of a thin film of polyethylene thermoregulate (PET), a thin layer of indium tin oxide was used as the first metallic track, and a nichrome alloy material (NiCr 80/20 wt) was deposited by electron beaming to generate four piezo resistors. Sensitivity values we reported at 6.365 mV/kPa. Hosseini et al. [21] reported a piezoelectric pressure sensor based on free-standing biodegradable piezoelectric films containing glycine—chitosan. It showed a sensitivity of 2.82 mV/kPa.

Accurate atmospheric pressure measurements are vital for weather forecasting, aviation, and climate studies. Sensors capable of detecting minute pressure changes contribute significantly to these fields, as seen in Sonlist [22]. In microfluidic devices and lab-on-a-chip, controlling fluid flow and pressure on the micro scale requires high-sensitivity sensors to detect small pressure differences accurately, as seen in Kurup et al. [23].

In this work, we present a novel large-diameter EFPI pressure sensor design achieving unprecedented 3.35 mPa accuracy with a sensitivity of 149 nm/kPa. A key innovation in our sensor design is the significantly larger diaphragm diameter (800 μm) compared to conventional sensors (typically around 125 μm), which fundamentally enhances the pressure-sensing capabilities. We explore innovative fabrication methods combining precision glass processing techniques with additive manufacturing approaches. Our research addresses specific challenges in diaphragm fabrication with the investigation of manual polishing, automated reduction, and chemical etching techniques.

These advancements in sensor performance and fabrication methodology establish a foundation for next-generation pressure-sensing technologies capable of detecting the smallest pressure variations in applications ranging from weather forecasting to lab-on-chip microfluidic control systems and medical devices, where precisely monitoring minute pressure differences is crucial.

### Table of Comparative Sensors

Shown below in Table 1 is a comparative table of FPI pressure sensors.

Wu et al. demonstrated an 11 μm thick 3D-printed diaphragm with a diameter of 80 μm and a ceramic ferrule, achieving a sensitivity of 3.3 ηm/kPa.

Other materials have been investigated, such as Cheng et al. silk fibroin, and Zhang et al.’s epoxy adhesive film. Other silica diaphragms have been constructed with diaphragms similar to the diameter of an SMF fibre, such as Donlagic and Cibula, whilst Duraibabu et al. demonstrated an underwater pressure sensor with a sensitivity of 15 ηm/kPa and a resolution of 78 Pa with a 130 μm diameter at 2 to 3 μm thick.

## 2. Sensor Theory and Design

### 2.1. Mechanical Design Considerations

The sensitivity of a diaphragm can increase dramatically as its diameter increases, the reason being a fourth-power relationship. Roark’s formula states that the centre deflection of a circular diaphragm of uniform thickness with clamped edges experiencing uniform pressure is given by [27],

(1) $$S_{\mathrm{linear}}=\frac{w}{P}=\frac{3(1-\nu^{2})}{16}\;\cdot \;\frac{a^{4}}{Et^{3}}$$

where *a* is the radius of the diaphragm, *t* is the thickness of the diaphragm, *E* is the Young’s modulus, and ν is the Poisson’s ratio.

When the diaphragm transitions from linear to non-linear, the non-linear load-deflection relationship for a clamped circular diaphragm under uniform pressure is given by,

(2) $$P=A\omega +B\omega^{3}$$

where *P* is the applied pressure, *w* is the central deflection, $A=\frac{\left(3+\nu \right)Et^{3}}{a^{4}}$ is the linear stiffness term, and $B=\frac{(1-\nu )Et}{a^{2}}$ is the non-linear term (membrane).

The local differential sensitivity (the mechanical response of the diaphragm at a given pressure) is defined as.

(3) $$\frac{dw}{dP}=\frac{1}{A+3Bw^{2}}$$

**Figure 1:**
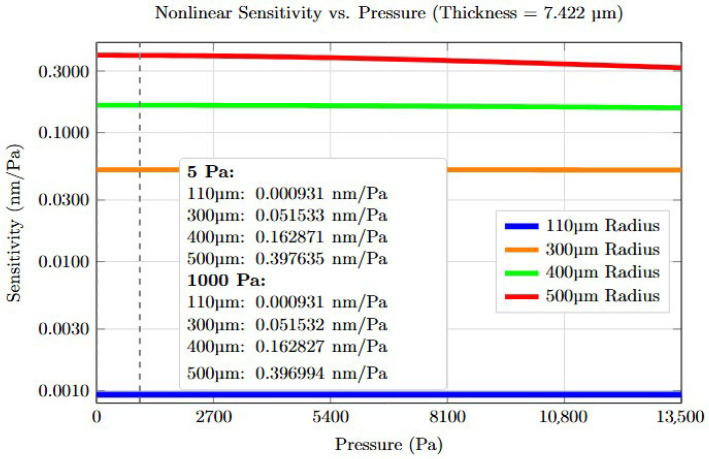
Non-linear sensitivity comparison for circular diaphragm pressure sensors with radii of 110 μm (blue), 300 μm (orange), 400 μm (green), and 500 μm (red). All configurations: thickness = 7.4 μm, Young’s modulus = 70 GPa, Poisson’s ratio = 0.17. Vertical dashed lines indicate reference pressures at 5 Pa and 1000 Pa.

The sensitivity increases by a factor of more than 1200 when a sensor with a diaphragm diameter of 125 μm is increased to a diameter of 800 μm. Figure 1 shows the difference in sensitivity plots for diameter of 220, 600, 800, and 1000 μm from 0 to 13,500 Pa.

The deflection ω can be calculated using Young’s modulus (E) and Poisson’s ratio (ν); (a) is the radius of the diaphragm and (t) is the thickness of the diaphragm. The pressure differential will result in a diaphragm deflection and, as such, a change in the cavity length of the sensor (w). In order to determine the diaphragm responses, the determination needs to be established as to whether it is a linear or non-linear response. This is determined by the deflection ratio as shown in Equation (4),

(4) $$DeflectionRatio=\frac{w}{t}$$

When $\frac{w}{t}\ll 0.3$, bending dominates and the classical linear plate theory is valid.

When $\frac{w}{t}⩾0.3$, the system transitions from linear to non-linear bending, where membrane stresses and stiffness become significant [27],

(5) $$A=\frac{\left(3+\nu \right)Et^{3}}{a^{4}},\;B=\frac{(1-\nu )Et}{a^{2}}$$

The deflection can be calculated for a given pressure by solving the cubic Equation (6) for ω at a given pressure using the Newton–Raphson method.

(6) $$A\omega +B\omega^{3}-P=0$$

The sensitivity *S* for any given pressure can be calculated using Equation (7) [27],

(7) $$S\left(P\right)=\frac{\omega (P)}{P}$$

where S(P) is the sensitivity at a given pressure and ω(P) is the deflection at the given pressure.

The breaking pressure can be calculated using Equation (8) [28]:

(8) $$P_{\mathrm{break}}=\frac{4\;\sigma_{f}\;t^{2}}{3\left(1+\nu \right)a^{2}}$$

The maximum deflection can then be calculated by using $P_{break}$ as the *P* value in Equation (6).

In this study, we propose the use of the sensor throughout its full range that extends well beyond the limits of linear plate theory, that is, where the deflection-to-thickness ratio ω/t exceeds 0.3. After this pressure, the membrane tension introduces significant geometric non-linearity. Therefore, non-linear Roark is used throughout the entire range [27].

The process for manufacturing a large-core-diameter EFPI is difficult and has not been explored. The reason is that the obvious applicable geometry to utilize is based on a standard SMF, and as such, the commercial tooling that has been developed for single-mode fibres. As such, new processes and techniques were required for the development of large-diameter all-glass EFPIs.

### 2.2. Interferometer Theory

**Figure 2:**
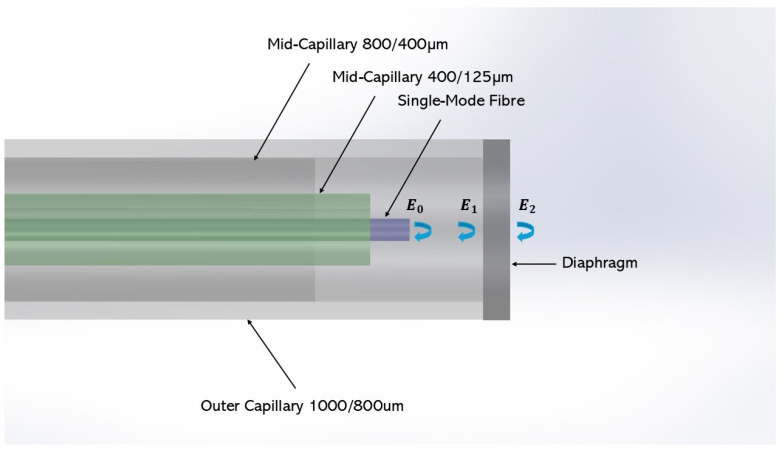
Schematic of the EFPI sensor.

The Fabry–Pérot Interferometer is grounded in the fundamentals of interferometry. The cavity geometry is from the single-mode fibre tip to the inner face of the diaphragm. It is housed in a glass capillary. The coherent light source enables interference between different optical paths within the sensor cavity. With respect to this sensor, approximately 4% of the light from the source through the SMF end face and the inner and outer faces of the diaphragm is reflected as seen in Figure 2.

It is worth noting that the path length of the light in will be 2L, where *L* is the cavity length. The air cavity will have a refractive index $n_{0}$ and will have a phase difference $\phi_{0}$, where in Equation (9) [19] the intensity I for each wavelength λ is dependent on the cavity length L, and as such, the diaphragm deflection is shown in Equation (9),

(9) $$I\left(\lambda \right)=I_{1}+I_{2}+2\sqrt{I_{1}I_{2}}\;\cdot \;\cos\left(\frac{4\pi \;\cdot \;L\;\cdot \;n_{0}}{\lambda }+\phi_{0}\right)$$

The reflected spectrum from the EFPI is is the sum of the reflected waves where I is the intensity of the reflected signals, $\vec{E_{0}}$, $\vec{E_{1}}$, and $\vec{E_{2}}$, as seen in Figure 2.

(10) $$I={\left(\vec{E_{0}}+\vec{E_{1}}+\vec{E_{2}}\right)}^{2}$$

where $\vec{E_{0}}$ is the electric field strength of the light reflected at the single-mode fibre end face, $\vec{E_{1}}$ is light reflected from the inner face of the diaphragm, and $\vec{E_{2}}$ is the light reflected from the outer face of the diaphragm Equation (10) [19]:

(11) $$I\left(\lambda \right)=E_{0}\;\cdot \;E_{1}\;\cdot \;\cos\left(\frac{4\pi n_{0}L}{\lambda }\right)+E_{0}\;\cdot \;E_{2}\;\cdot \;\cos\left(\frac{4\pi (n_{0}L+n_{l}d)}{\lambda }\right)+E_{1}\;\cdot \;E_{2}\;\cdot \;\cos\left(\frac{4\pi dn_{1}}{\lambda }\right)$$

where *L* is the sensor cavity length, $n_{0}$ is the refractive index of the sensor cavity medium and the external environment, $n_{1}$ is the refractive index of the sensor diaphragm, *d* is the diaphragm thickness, and λ is the wavelength [19].

### 2.3. Sensor Design and Fabrication

**Figure 3:**
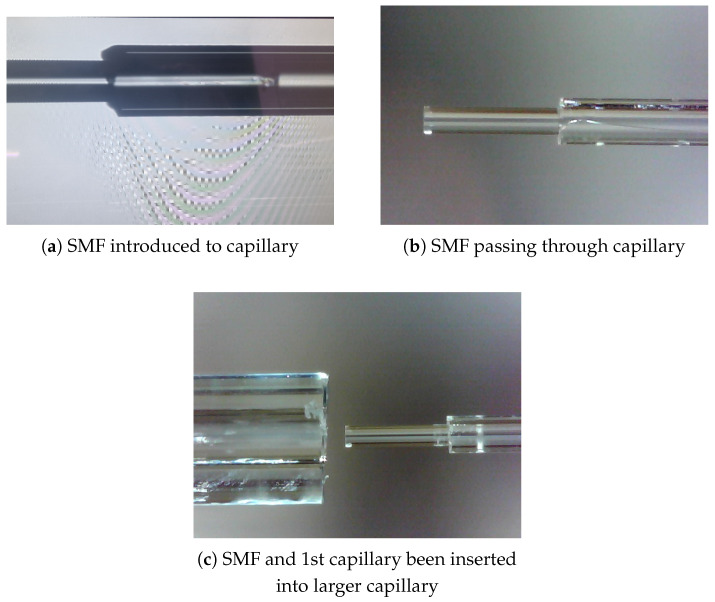
Inner sensor assembly components.

The sensor assembly fabrication consists of two primary steps. Firstly, the inner sensor assembly, which includes the single-mode fibre (SMF) and the surrounding capillaries that form the core structure, as illustrated in Figure 3a–c. Secondly, the outer sensor assembly which consists of a diaphragm coupled to an outer sleeve designed to enclose the inner sensor assembly and hermetically seal the Fabry–Pérot interferometer (FPI) cavity. For this outer assembly, both glued and spliced configurations are tested, ensuring flexibility in the sealing approach while maintaining structural integrity and functionality.

The fabrication process of the EFPI pressure sensors involved several steps to ensure precision and functionality. Initially, a single-mode fibre (SMF) was cleaved and stripped to a length of 125 mm. A capillary with an inner diameter of 135 μm and an outer diameter of 240 μm was then cleaved to a length of 115 mm, where an SMF was inserted into it using an Ericsson FSU 975 splicer sourced from Custom Calibration Solutions, Hamilton Township, NJ, USA (Figure 3a). The SMF, fed through the capillary, was carefully retracted to protect its tip. Subsequently, another capillary with an inner diameter of 245 μm and an outer diameter of 750 μm was cleaved and attached to the inner sensor assembly (Figure 3c). The capillaries were glued together without sticking the SMF to the inner capillary. The SMF was then extended through the capillary so that it protruded 100 to 200 μm beyond the inner capillary, with the smaller capillary extending approximately 200 μm beyond the larger capillary.

### 2.4. Outer Sensor Assembly with Adhesive

The first challenge was to determine a process to cleave and polish the 1 mm rod and the 1 mm capillary. A standard polishing apparatus cannot accommodate these large diameters.

#### 2.4.1. Polishing the 1 mm Capillary and Rod

**Figure 4:**
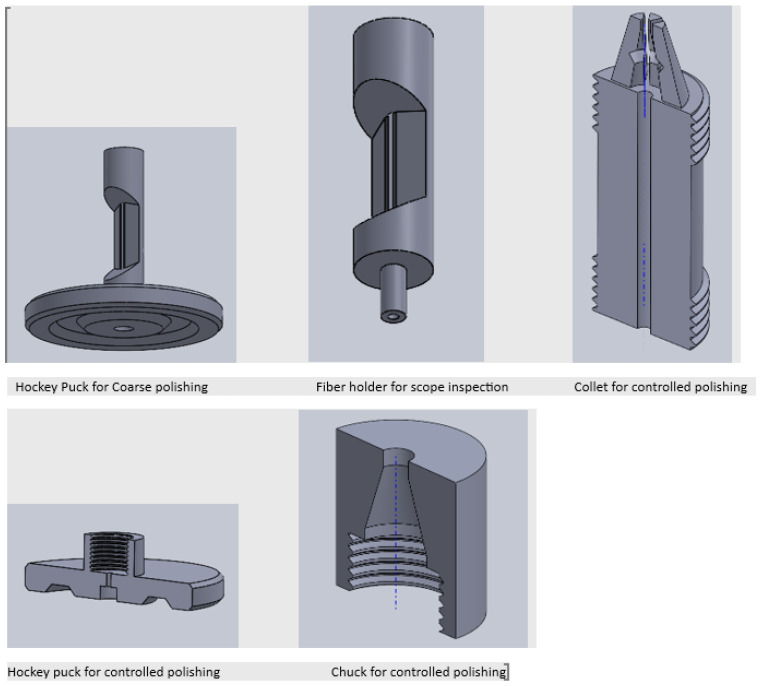
Selection of printed polishing assemblies.

As such, tooling capable of polishing 1 mm fibres was explored and was not applicable to large-diameter diaphragms. The solution lay in additive manufacturing/3D printing. This afforded an effective design to the manufacturing process. Figure 4 shows a series of images detailing the design and manufacturing process.

**Figure 5:**
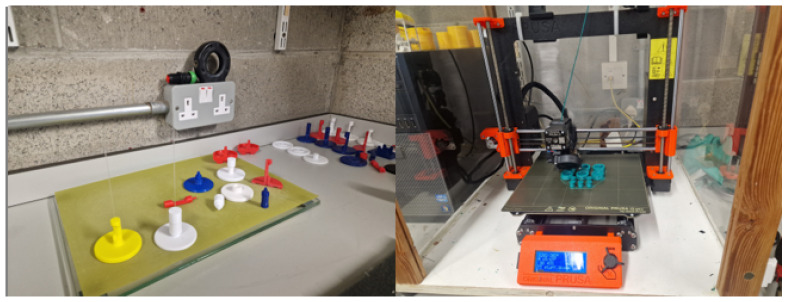
Polishing devices in use and during production.

The cleaving process was performed using a precision fibre scribe, followed by polishing with aluminium oxide, silicon carbide, and diamond lapping film down to a fineness of 0.05 μm as shown in Figure 5. This polishing was achieved using custom tooling, as illustrated in Figure 4.

#### 2.4.2. Alignment of the Rod and Capillary

To ensure proper alignment and secure connection between the diaphragm and capillary, a specialized mechanism was required. This mechanism utilizes two three-axis linear stages Purchased from USA Tools, Miami, FL, USA, equipped with custom 3d Printed 3D-printed adapters designed to fit OFS Fitel fibre holders (Model No’s: S712S-250, S712S-500, S712S-LT). These stages were mounted onto a rigid platform, precisely positioned to accommodate the capillary in one stage and a 1 mm rod in the other stage.

**Figure 6:**
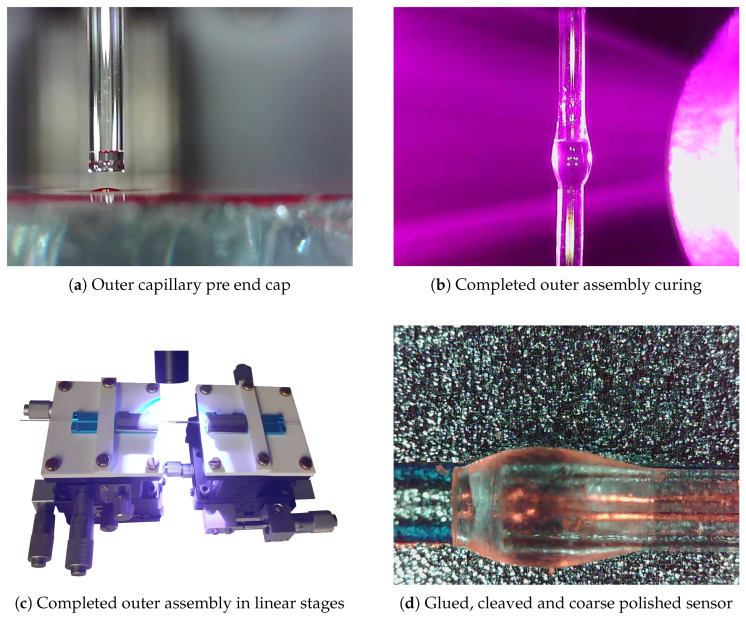
Glued Outer Assembly.

The end of the 1 mm rod and capillary were polished to a 0.5 μm surface finish using lapping film. The capillary was then inserted into the top linear stage, while a gluing platform was positioned on the lower stage. A thin, level film of glue was applied to the platform, as illustrated in Figure 6a. The capillary was introduced to the glue and subsequently removed, as shown in the figure. A sacrificial rod was carefully inserted through the top of the capillary and passed down through its lower opening to remove any residual glue from its interior. Subsequently, a 1000 μm rod was placed in the lower stage, introduced to the capillary, and cured with UV light, as shown in Figure 6b. Additional glue was applied to the outside of the joint and cured to ensure proper bonding, as shown in Figure 6c. Finally, the assembly was removed from the stages, the rod was cleaved to a length between 1 and 2 mm, and its thickness was polished to a range of 0.75 mm to 1 mm (Figure 6d).

### 2.5. Outer Sensor Assembly by Splicing

**Figure 7:**
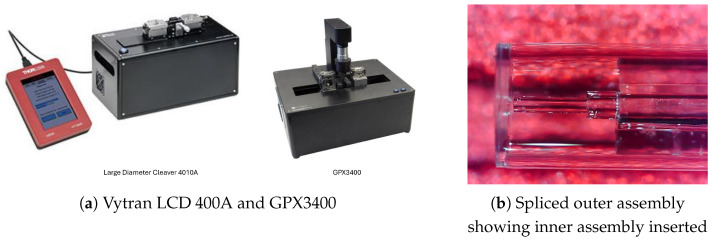
(**a**) GPX3400-Vytran Automated Glass Processor and an LDC400A-Vytran Large-Diameter Cleaver. (**b**) Spliced outer assembly showing inner assembly.

This was carried out using Thor Labs GPX3400 Vytran Automated Glass Processor workstation and a LDC400A Large-Diameter Cleaver with Backstop, both from from Thor Labs, Exeter, UK as seen in Figure 7a with the spliced outer assembly shown in Figure 7b.

The first step was to configure the LDC400A. The bare 1 mm End cap was stripped of its jacket, cleaned with acetone, and wiped down with IPA. The circumference of the rod was scribed with 120 peak cycles. The tension was set to 4305 *G*, where it was cleaved. The capillary was then cleaved at 1400 *G*. The cleaves are then assessed, and if the cleave angles are high or any damage is present, then new parts are prepared. The parts are introduced to the GPX3400, where they are spliced. The RHS side, which contained the 1000 μm rod, was released; then, the LHS was released, but the transfer case was closed. The spliced assembly was then moved back to the LDC400A, where the offset for the end cap cleaving was set to 400 μm, and it was then cleaved under a tension of 2000 *G*.

The cleaving results for the capillary varied and remained inconsistent through various tension settings. However, enough samples were obtained in the process. The end cap cleaving was mostly fine, but some of the assemblies failed under tension adjacent to the splice. Once again, enough samples were obtained.

### 2.6. Assembling the Complete Sensor

**Figure 8:**
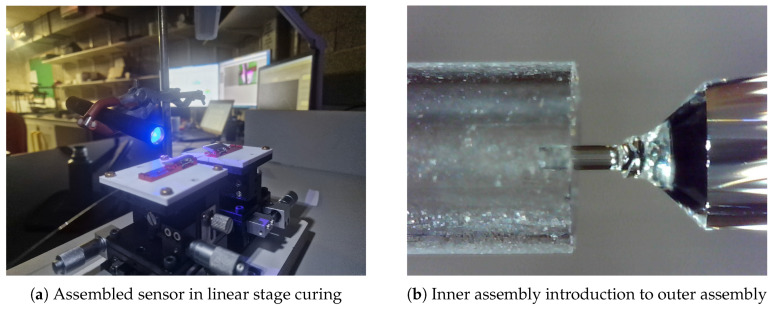
Inner assembly introduction to outer assembly.

With the inner and outer sensor assembly now complete, it could be assembled. Both the inner and outer assemblies were placed on opposing linear stages, and the IA was introduced to the OA. Once alignment was confirmed, some adhesive was added, and the IA was inserted into the OA until the end of the SMF on the IA was within circa 50 microns from the inner face of the OA. The distance of the SMF from the diaphragm was monitored using a microscope to 184 ensure optimal positioning. The spectral response of the cavity was observed, and once the correct length was achieved, the assembly was cured using UV light. The completed sensor can be seen in Figure 7b and Figure 8a.

## 3. Assembled Sensor Diaphragm Reduction

### 3.1. Reduction with Polish and Lapping Film

The tooling developed in Figure 4 was used to further reduce the diaphragm from ≈500 μm to sub 40 μm. Aluminium oxide, silicon carbide, and diamond lapping film from 12 μm to 0.05 μm grade were utilized.

**Figure 9:**
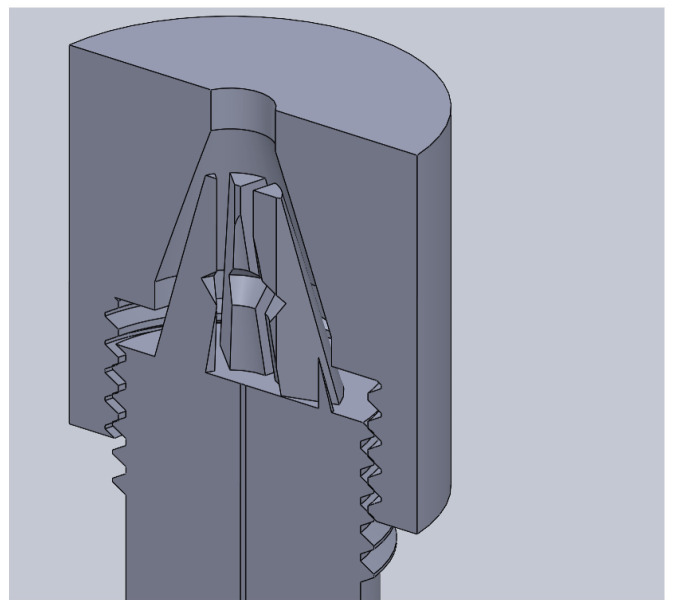
Sectional collet and clamp nut assembly.

The first design challenge was to design a mechanism capable of sensor insertion and removal which would have the of securing securing the sensor without crushing it within the polishing tool. It was determined that a collet-type mechanism, as shown in Figure 9, should be designed and manufactured.

**Figure 10:**
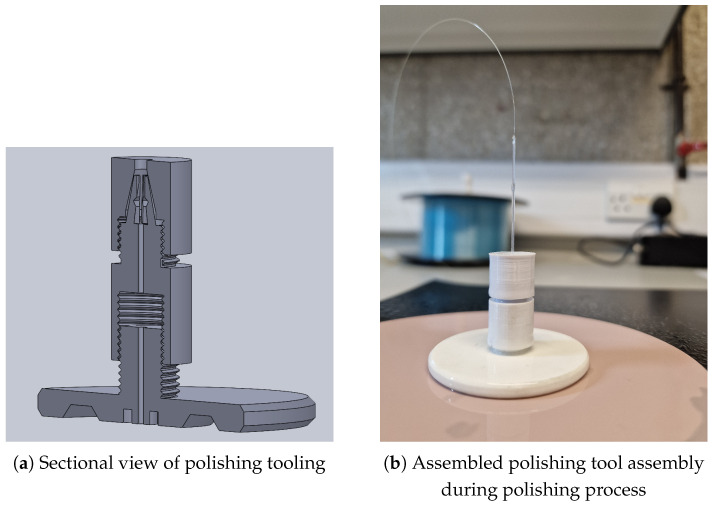
Assembled diaphragm reduction tooling.

The next obstacle was to design a system to introduce the sensor diaphragm to the polishing film in a controlled manner. This was achieved using a mortice-and-tenon-style mechanism, which could be joined with the above assembly. After multiple iterations in conjunction with polishing trials, the mechanism shown in Figure 10a,b was selected.

**Figure 11:**
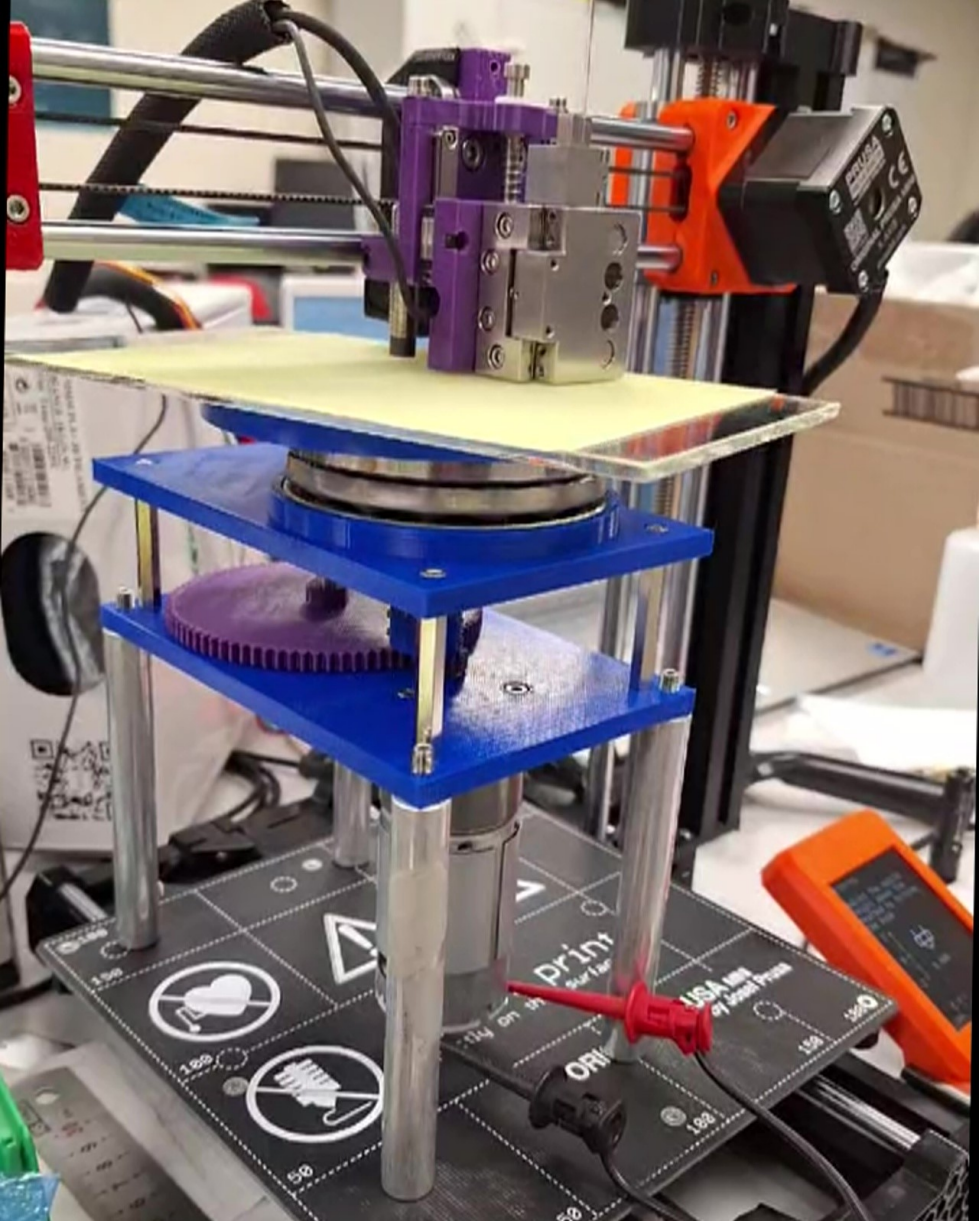
Automated diaphragm reduction.

The other technique trialled was an automated solution utilizing a 3D printer, as shown in Figure 11; this, however, proved not to have the sensitivity required and failed at 50 μm. The limitations of the process were attributed to the resolution and indeed the sensitivity of the stepper motors, which, with 200 steps per 0.8/mm, gave a vertical displacement height of 4 μm. This is the theoretical sensitivity; however, with backlash in the gearing system, the limitation of 50 μm is a respectable outcome. It is this which, in turn, determines the force with which the polishing film interacts with the outer diaphragm face.

The subsequent challenge pertained to the characterization and examination of the sensor diaphragm during the polishing and reduction process. The fundamental operating principle of the sensor is based on a variation in cavity length induced by diaphragm flexure. However, as the diaphragm undergoes reduction, its optical properties are altered, resulting in a shift in the spectral response. This spectral response, combined with imaging facilitated by a microscope, constituted the primary method for evaluating and refining the sensor geometry during the reduction process. Diaphragm deflection was observed as a phase shift when the thickness of the diaphragm decreased below 50 μm.

Visual examination of the diaphragm was initially not feasible due to the absence of tooling jigs and fixtures compatible with the sensor housed within the clamping mechanism. Consequently, the structural integrity of the diaphragm was evaluated exclusively by the spectral response of the sensor.

The initial batch of sensors was polished under these constraints, achieving diaphragm thicknesses of approximately 50 μm. Although these diaphragms exhibited sufficient flexural responses, they failed pressure calibration tests. The results indicated that the sensors were not hermetically sealed. With the structural integrity of the diaphragm confirmed through spectral analysis, the adhesive utilized to seal the inner and outer sensor assemblies was identified as a potential failure point. The adhesive was subsequently resealed; however, subsequent tests continued to reveal pressure equalization between the sensor cavity and the external environment. Following the elimination of the adhesive as the failure mechanism, it became evident that a methodology was imperative to visually examine the diaphragm during the polishing process. The development of appropriate jigs and fixtures was prioritized to allow a continuous evaluation of the structural integrity of the diaphragm throughout successive polishing iterations. It should be noted that the sensor assembly remained securely housed within the clamping mechanism throughout the process.

**Figure 12:**
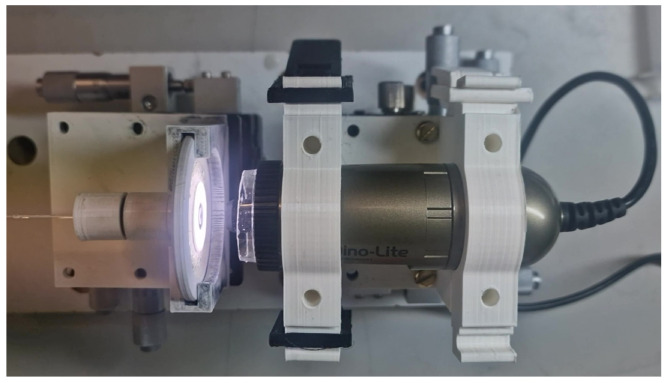
Diaphragm examination.

Eventually, additive manufacturing became a critical solution to addressing these challenges. Opposing three-axis linear stages were used in conjunction with 3D-printed jigs and fixtures, facilitating precise evaluation of the diaphragm by digital microscopy, as illustrated in Figure 12.

**Figure 13:**
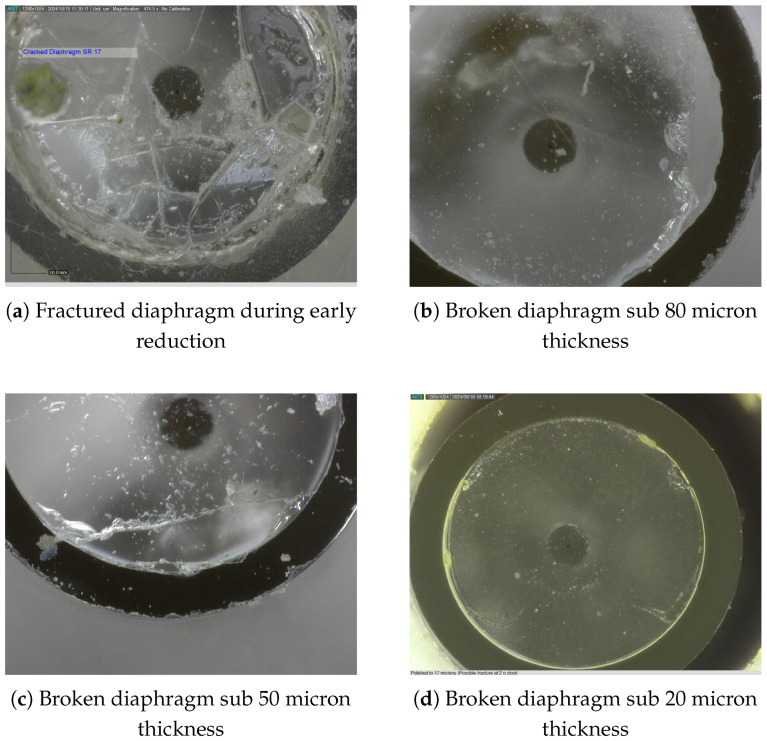
Compromised diaphragms during final polish.

The compromised sensors were examined, and the results revealed, as shown in Figure 13, that whilst the diaphragms were flexing during the final polishing process and with sudden pressure changes, they were fractured.

**Figure 14:**
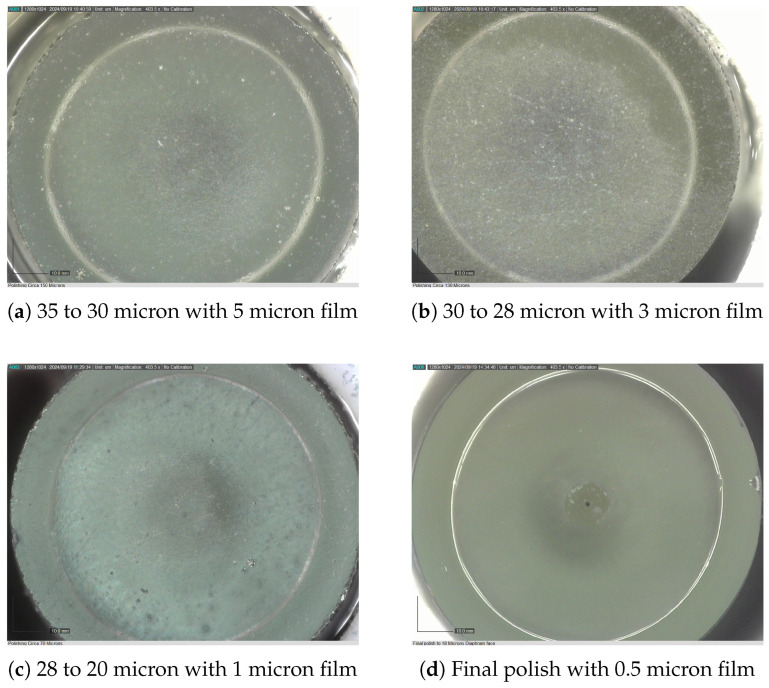
Fully polished sensor Circa 20 μm.

Equipped with advanced imaging capabilities, the polishing methodology was refined to achieve diaphragm thicknesses below sub 30 μm with robust diaphragm integrity. Figure 13 illustrates the diaphragms compromised during the final stages of polishing, alongside the progressive refinement of the polishing process. This refinement ultimately enabled the production of robust sensor diaphragms as depicted in Figure 14a–d.

**Figure 15:**
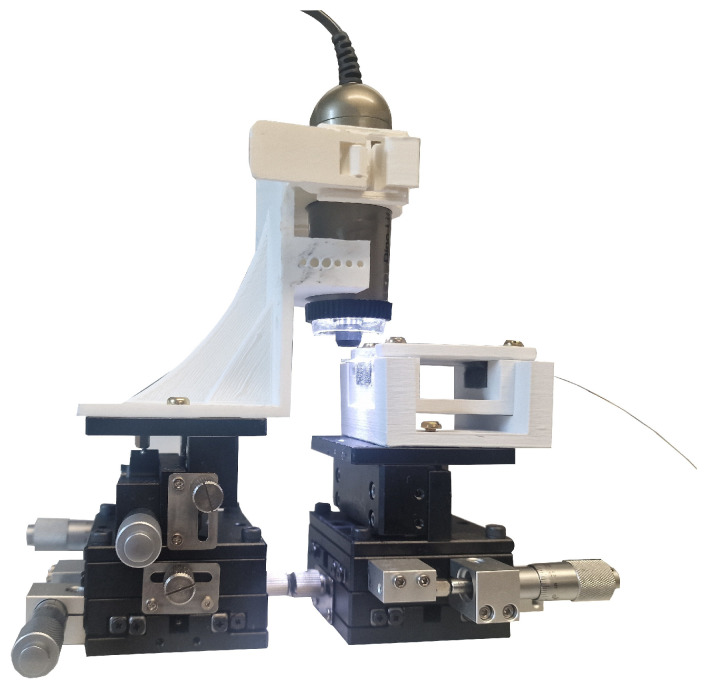
Visual determination of diaphragm thickness.

To accompany the face in view, a microscope and sensor mounting system was developed to visually determine the thickness of the diaphragm via a perpendicular scope, as shown in Figure 15.

**Figure 16:**
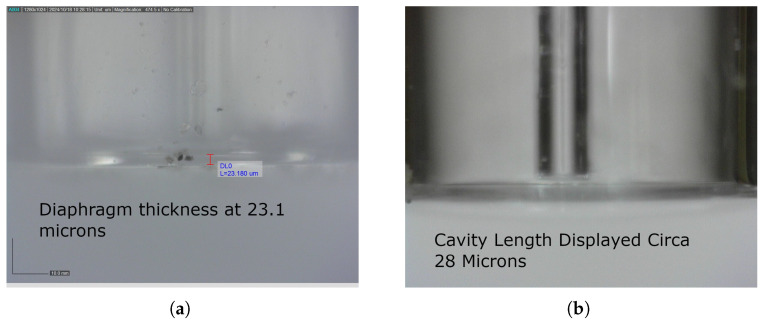
Sensor geometry. (**a**) Diaphragm thickness. (**b**) Cavity length.

Figure 16a displays the diaphragm thickness, and Figure 16b displays the cavity length.

#### Final Reduction with HF Etching

**Figure 17:**
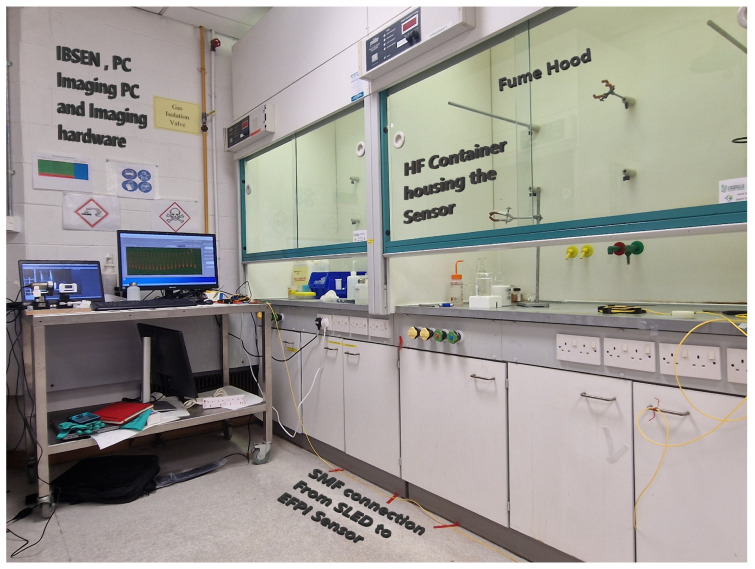
Fume hood layout.

The diaphragm was further reduced using hydrofluoric (HF) as a reducing agent in an etching process. The sensor end was immersed in the solution. This process took place inside a fume hood in a controlled environment, as shown in Figure 17.

**Figure 18:**
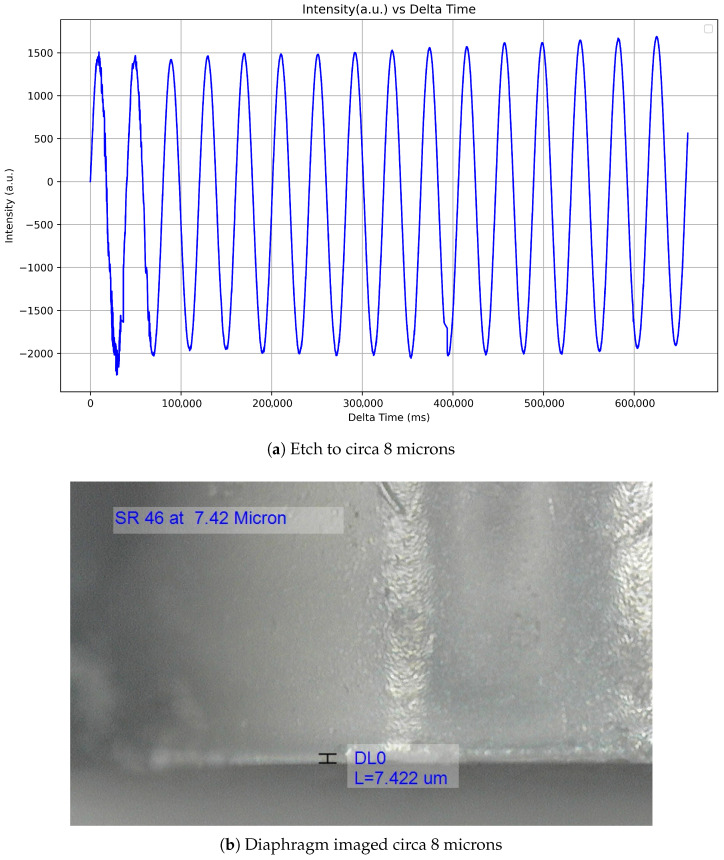
Etching process and final diaphragm. (**a**) Periodic change in spectrum during the etch process. (**b**) Diaphragm at 7.4 μm.

The etching process and, as such, the diaphragm reduction were monitored through changes in the reflected spectrum. Analysis of the Q-point within the interference pattern in the spectrum revealed that a periodic change corresponds to a reduction of 0.5 μm in the thickness of the diaphragm, as seen in Figure 18a. The selected Q-point is positioned within the linear region of the baseline interference pattern, specifically near the midpoint between a peak and a trough in the spectral response.

When the calculated desired diaphragm geometry is achieved, the diaphragm is removed from the solution, and the sensor is then immersed in distilled water to remove any residual hydrofluoric acid solution. Then, it is imaged using the Dinolite microscope [29], as seen in Figure 18b.

## 4. Experimental Setup

### 4.1. Optical Interrogation Setup

**Figure 19:**
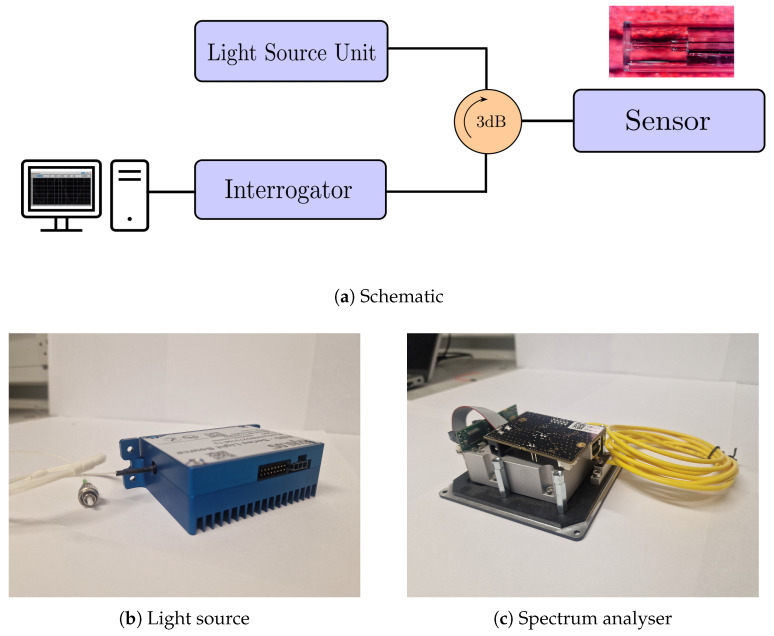
(**a**) Optical interrogation setup. (**b**) EXALOS light source. (**c**) Optical spectrum analyser.

An EXALOS EBD2720003-03 broadband light (BBL) from Exalos source sourced in Schlieren, Switzerland unit (Figure 19b) [30] generated a Gaussian light output with a bandwidth of 45 μm centred around at 1550 nm. The optical power was 10 to 15 mW. It propagated through the 3 dB coupler and on to the EFPI sensor. The modulated signal was then returned through the SMF through the 3 dB coupler to the OSA Ibsen (I-MON-512E) manufactured by IBSEN from Farum, Denmark (Figure 19c) [31]. This, in turn, utilized a linear GaAs image sensor with 512 pixels, having a wavelength fit resolution of <0.5 pm form Δλ from 1510 to 1595 nm. This, in turn, interfaced with a PC and ran on a LabVIEW™ app licenced to University of Limerick, Ireland that interrogated the signal. The following components were utilized in the interrogation setup and are illustrated in Figure 19a.

### 4.2. Experimental Setup

**Figure 20:**
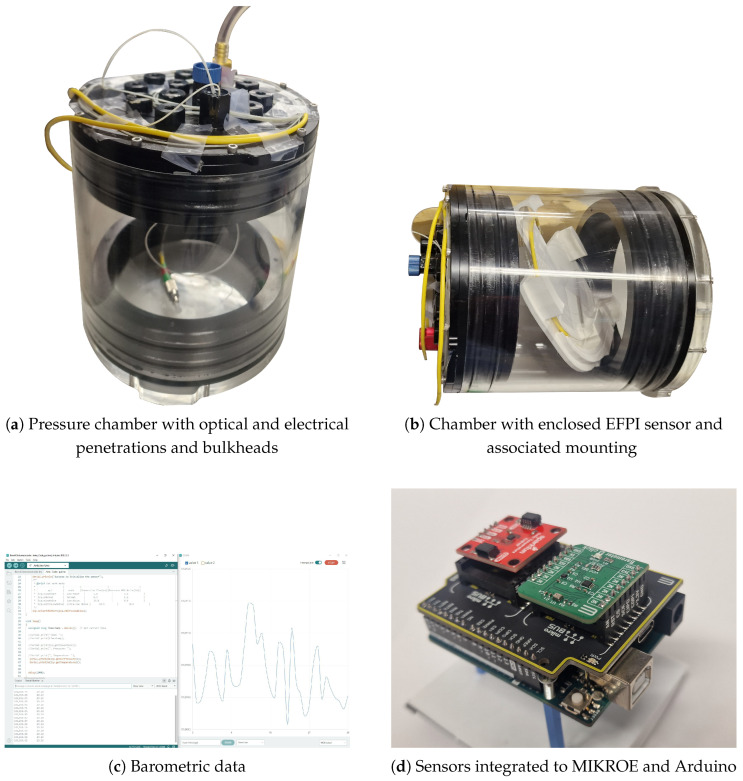
Pressure testing and calibration in pressure housing. (**a**) Raw sensor housed in chamber. (**b**) Sensor enclosed in chamber. (**c**) Barometric sensor code. (**d**) Electronic SR suite.

The sensor calibration was conducted using a high-accuracy, low-power barometric and temperature sensor, the TDK InvenSense ICP-10111 [32] integrated onto a Mikroe 4 click board sourced from Mikroe (Belgrade, Serbia) [33], and interfaced with an Arduino UNO Sourced from Radionics (Dublin, Ireland) [34], as illustrated in Figure 20d. This sensor offers an accuracy of ±1 Pa, a measurement range of 30 to 110 kPa, and a resolution of 0.01 Pa.

The EFPI sensor was enclosed within a hermetically sealed chamber, as depicted in Figure 20a in an anechoic chamber. The single-mode fibre (SMF) of the EFPI sensor was routed into the chamber through a Blue Robotics bulkhead penetrator, sourced from Blue Robotics California USA shown in Figure 20b. The penetrator included a venting port to allow the chamber to be exposed to or isolated from ambient atmospheric pressure as required.

The pressure chamber and the incorporated sensors were exposed to atmospheric pressure. Subsequently, the chamber was sealed, and pressure was recorded at the time of sealing. The EFPI sensor was interrogated using the optical architecture shown in Figure 19a, with the corresponding hardware arrangement shown in Figure 20.

## 5. Results and Discussion

**Figure 21:**
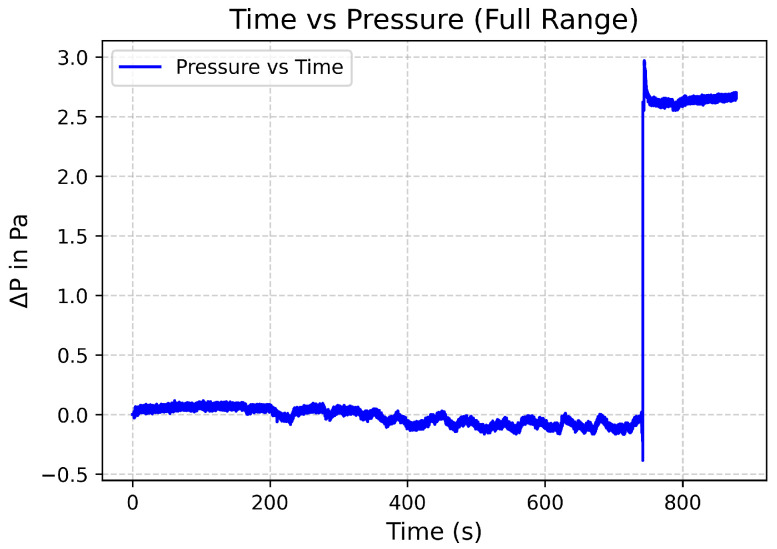
Plot of pressure differential of 2.7 Pa.

**Figure 22:**
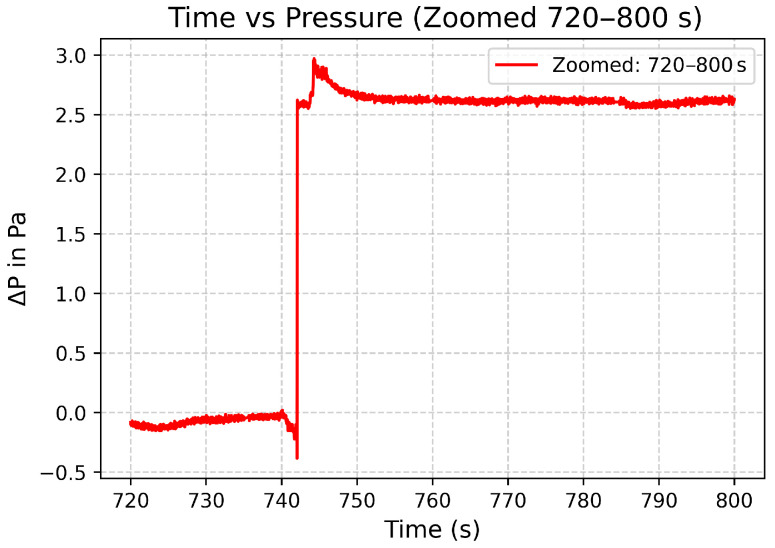
Zoomed in plot of pressure differential of 2.7 Pa.

The sensor yielded a sensitivity of 149 nm/kPa and a resolution of 3.35 mPa. The data can be seen in Figure 21 and Figure 22.

The glass construction of the sensor, particularly the diaphragm, has its challenges. The failure rate during sensor construction and diaphragm reduction is significant. All glass sensors are successfully produced and demonstrate repeatable and reliable results [35].

**Figure 23:**
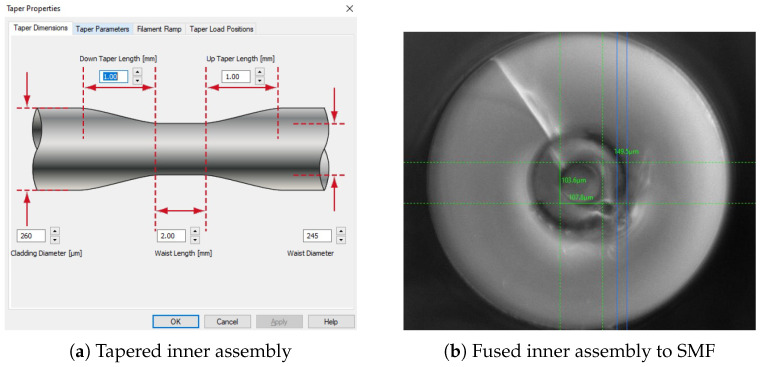
(**a**) Tapered inner assembly A. (**b**) Fusing of tapered IA to SMF.

The authors are of the opinion that the process will benefit from the removal of adhesive from the construction process and the elimination of multiple inner assembly capillaries. Initial experiments were carried out with the coupling and splicing of a single inner capillary ID 250 μm OD 800 μm to the SMF (Figure 23b). Fusion of the OA with the tapered IA may be a viable option, as shown in Figure 23.

**Figure 24:**
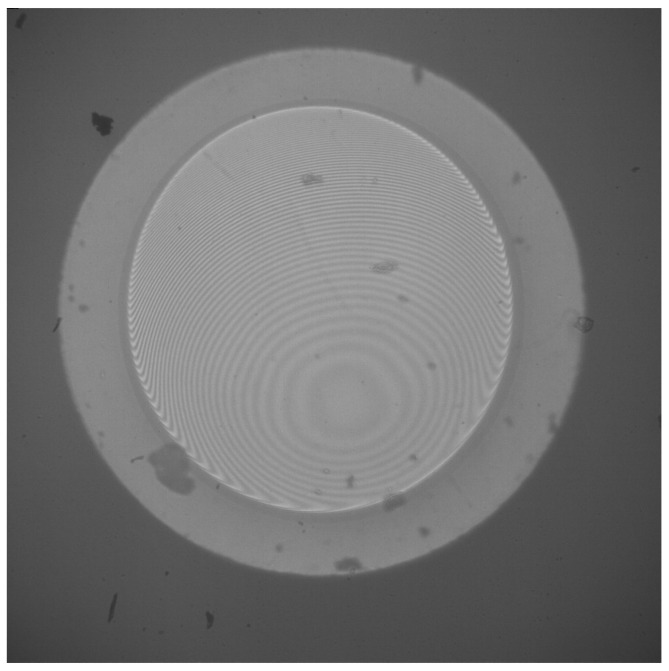
Innerdiaphragm geometry.

The splicing process of the diaphragm onto the outer capillary facilitates the flow of glass onto the inner face of the capillary as seen in Figure 24. This may be significantly reduced by utilising an iridium or tungsten filament rather than a graphite filament, as the heating ramp-up time prior to splicing will be greatly reduced. However, there will always be some flow! Every sensor will differ, and the inner and outer faces of the diaphragm will need to be imaged and modelled using both the cleave analyser and interferometry for the inside face and photogrammetry for the outer face, in order to correctly determine each individual sensor’s mechanical and optical properties.

The cleaving process can only be as small as 50 microns, as the face will not be even. The reason for this is that the force necessary to carry out a perpendicular cleave is more than the tensile force that the spliced outer capillary/diaphragm can withstand. Therefore, an automated diaphragm reduction technique will be developed.

The variation in the internal angle of the diaphragm will affect the sensor response. Structurally the diaphragm response can be evaluated using Young et al. [27] (Section 11.2, pp. 441–442) The interferometric pattern will change, as the cavity length will decrease towards the perimeter of the diaphragm, whereas the diaphragm thickness will increase towards the perimeter of the diaphragm.

Every sensor will need to be modelled using finite element analysis, as both the etched surface roughness and the inner surface of the diaphragm will differ for every sensor produced.

## 6. Conclusions and Future Development

In this paper, the results achieved with the EFPI demonstrated significant improvements with both the sensitivity and resolution of the associated EFPI, with a resolution of 3.35 Pa and a sensitivity of 149 nm/kPa. It can be seen that when an Arden cleave analyser, as shown, is used to model the inner face of the diaphragm, Figure 24 shows an angle of up to 1.2°. The determination is that this could be due to the splice process that facilitates the flow of glass into the capillary. This in itself could lead to a variance of up to 8.4 μm on the inside face alone.

The diaphragm reduction technique is labour-intensive, detailed, and subject to failure. Automated techniques such as the PRUSA 3D printer arrangement were explored, as shown in Figure 11.

Various sensor diaphragm reduction tools such as shown in Figure 4 and Figure 5 were developed.

During the latter stages, the diaphragm polishing the diaphragm deflects as it experiences an upward force from the polishing paper. This force is monitored through the LabVIEW software referenced earlier, as a cavity length decline and as such a spectral shift. This response can be integrated into the future automated diaphragm control architecture.

**Figure 25:**
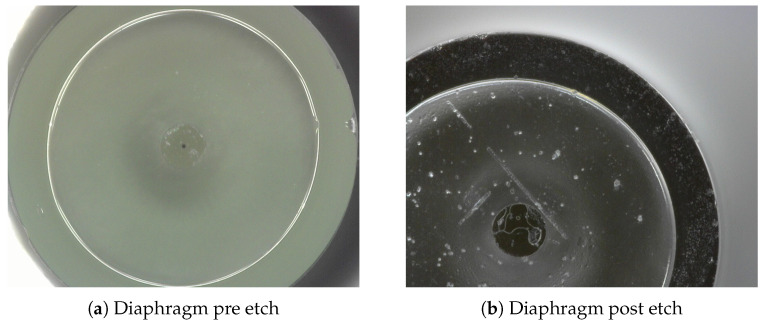
Diaphragm pre and post Etch.

The HF wet etching diaphragm technique will inevitably lead to surface roughness. Sun et al. reported surface roughness of up 0.59 nm reported for etch depths of more than 3 μm [36]. Chen [37] also reported that whilst the concentration of HF determines the etch rate, a lower concentration of HF will result in a smoother surface finish post-etch. Below are images of the diaphragm pre- and post-etch. Future research with machine vision and CAD modelling will be able to determine the impact of such surface roughness on the diaphragm post HF etching compared to pre-etch as seen below in Figure 25.

### Long Term Stability

When we consider the long-term stability of the sensor, the objective moving forward is to develop an automated manufacturing process where fully fused all-silica construction will be utilized, rather than the current fused silica and adhesive construction. This will align all the mechanical and thermal properties of the sensor, as it will be composed solely of one material with proven long-term stability; we will also integrate an FBG into the inner sensor assembly prior to sensor assembly [38].

## Figures and Tables

**Table 1:** Comparative table of sensors.

Sensor Construction and Materials	Ref	Fabrication Method	Pressure Range (kPa)	Sensitivity (nm/kPa)	Resolution (Pa)	Diaphragm Diameter (Microns)	Diaphragm Thickness (Microns)
All-fibre high-sensitivity pressure sensor	[17]	Fusion splicing, Etching, Polishing		3.4	300	125	
SMF, MM diaphragm & silica capillary	[19]	Fusion splicing, Wet polishing & Etching		15	78	130	2–3
SMF, Capillary & Epoxy adhesive film	[5]	Epoxy Adhesive and Fusion splicing	0–70	0.257		150	8.74
Single-mode fibre, silica capillary and Diaphragm Fusion splicing and etching.	[24]	Silica Capillary, Diaphragm and SMF		1–1.6	28	130	2.3
Silk Fibroin, Silica Capillary	[25]	Epoxy adhesive to bond the Capillary to the SMF& aminopropylitrieethoxysilane on the end face of the capillary to bond to the Fibroin diaphragm		12.3		127	20
SU-8 epoxy Polymer diaphragm, SMF, Ceramic ferrule	[26]	3D Printing	0–700	0.00293		80	11

## Data Availability

The raw data supporting the conclusions of this article will be made available by the authors on request.

## Abbreviations

The following abbreviations are used in this manuscript:

FPI	Fabry–Pérot Interferometer
EFPI	Extrinsic Fabry–Pérot interferometry
HF	Hydrofluoric Acid
ISA	Inner Sensor Assembly
OSA	Outer Sensor Assembly
FSV	Full-Scale Value
MMF	Multimode Fiber
SMF	Single-Mode Fiber
IA	Inner Assembly
OA	Outer Assembly
ID	Inner Diameter
OD	Outer Diameter
RHS	Right-Hand Side
LHS	Left-Hand Side
UV	Ultra Violet
OFS	OFS Optics
LDC	Large-Diameter Cleaver
OSA	Optical Spectrum Analyser
BBL	Broadband Light Source
PC	Personal Computer
SNR	Signal-to-Noise ratio
SR	Sensor
Pa	Pascal
